# Trabalho, saúde mental, qualidade de vida e suporte social de agentes comunitários de saúde durante e pós-pandemia de COVID-19 sob o recorte de gênero

**DOI:** 10.1590/0102-311XPT168824

**Published:** 2025-07-04

**Authors:** Grayce Alencar Albuquerque, Regina Glaucia Lucena Aguiar Ferreira, Andréa Silvia Walter de Aguiar, Isabella Lima Barbosa Campelo, Maria Socorro de Araújo Dias, Anya Pimentel Gomes Fernandes Vieira-Meyer

**Affiliations:** 1 Universidade Regional do Cariri, Crato, Brasil.; 2 Universidade Federal do Ceará, Fortaleza, Brasil.; 3 Instituto Materno Infantil do Ceará, Fortaleza, Brasil.; 4 Centro Universitário UniFanor, Fortaleza, Brasil.; 5 Universidade Estadual Vale do Acaraú, Sobral, Brasil.; 6 Fundação Oswaldo Cruz, Eusébio, Brasil.

**Keywords:** Agentes Comunitários de Saúde, Saúde Mental, Qualidade de Vida, Identidade de Gênero, Pandemics, Community Health Workers, Mental Health, Quality of Life, Gender Identity, Pandemias, Agentes Comunitarios de Salud, Salud Mental, Calidad de Vida, Identidad de Género, Pandemias

## Abstract

Esse estudo objetivou avaliar a saúde mental, qualidade de vida, suporte social e atividades desenvolvidas por agentes comunitários de saúde (ACS) durante e após uma crise sanitária, sob o recorte de gênero. Estudo multicêntrico, quantitativo, longitudinal, realizado em oito cidades do Nordeste brasileiro, nos anos 2021 e 2023, com 705 ACS pareados por meio do *propensity score matching*, que responderam a questionários validados, analisados sob o recorte de gênero (masculino e feminino), tempo (durante e após a pandemia de COVID-19) e interação entre estes. Efeito do tempo foi observado frente à violência nos territórios, atividades desenvolvidas, ansiedade para o coronavírus, saúde mental e qualidade de vida. Identificou-se efeito do gênero na redução do suporte social (amigos) em ACS mulheres e maior realização de visitas domiciliares e atividades do Programa Saúde nas Escolas por ACS homens. Interação entre gênero e tempo foi observada por meio da redução de violência contra ACS do gênero feminino e aumento no gênero masculino entre os tempos estudados. Embora homens e mulheres compartilhem os mesmos dilemas e dificuldades no exercício da função ACS, recai sobre as profissionais femininas as implicações do gênero, havendo neste público, maior desgaste físico e emocional e maior susceptibilidade ao adoecimento, especialmente em momentos de crises sanitárias.

## Abstract (en)

This study aimed to evaluate the mental health, quality of life, social support, and activities by community health workers (CHW) during and after a health crisis under a gender perspective. This multicenter, quantitative, longitudinal study was carried out in eight municipalities in the Brazilian Northeast in 2021 and 2023 with 705 CHW who were paired by *propensity score matching*, answered a validated questionnaire, and were analyzed under the focus of gender (men and women), time (during and after the COVID-19 pandemic), and interactions between them. The effect of time stemmed from the violence in the territories, developed activities, anxiety about the virus, mental health, and quality of life. The effect of gender stemmed from the reduction of social support (friends) in female CHW and greater performance of home visits and activities of the health in schools program by CHW. The interaction between gender and time stemmed from the reduction of violence against female CHW and an increase in the male gender between the studied periods. Although men and women share the same dilemmas and difficulties in exercising CHW, gender implications fall on female professionals, who show greater physical and emotional exhaustion and susceptibility to illness, especially in times of health crises.

## Abstract (es)

Este estudio tuvo como objetivo evaluar la salud mental, la calidad de vida, el apoyo social y las actividades desarrolladas por los agentes comunitarios de salud (ACS) durante y después de una crisis sanitaria bajo la perspectiva de género. Se trata de un estudio multicéntrico, cuantitativo, longitudinal, realizado en ocho ciudades del Noreste de Brasil en los años 2021 y 2023, con 705 ACS emparejados mediante *propensity score matching*, que respondieron cuestionarios validados, analizados bajo el género (masculino y femenino), tiempo (durante y después de la pandemia de la COVID-19) e interacción entre ellos. Se observó efecto del tiempo frente a la violencia en los territorios, en las actividades desarrolladas, en la ansiedad por el coronavirus, en la salud mental y en la calidad de vida. Se identificó el efecto del género en la reducción del apoyo social (amigos) en las mujeres ACS y mayor desempeño de las visitas domiciliarias y actividades del Programa de Salud Escolar por parte de los ACS hombres. Se observó interacción entre género y tiempo mediante la reducción de la violencia contra ACS del género femenino e incremento en el género masculino entre el período estudiado. Aunque hombres y mujeres comparten los mismos dilemas y dificultades en el ejercicio de la función de ACS, las implicaciones de género recaen en las mujeres profesionales al presentar mayor desgaste físico y emocional y mayor susceptibilidad a la enfermedad, especialmente en tiempos de crisis sanitarias.

## Introdução

Crises sanitárias e humanitárias tem sido observadas ao longo da história ^1^. Neste contexto se destacam as pandemias, que impõem grande impacto social, elevando índices de morbimortalidade da população e sobrecarga dos sistemas de saúde ^2^. 

Apesar do cenário de adoecimento desconhecido e coletivo gerado pela pandemia de COVID-19 e diante das dificuldades enfrentadas, da ausência de uma coordenação nacional, das diferenças regionais e da heterogeneidade do processo de trabalho das equipes de atenção primária à saúde (APS), o Sistema Único de Saúde (SUS) conseguiu atuar no enfrentamento da pandemia de COVID-19 ^3^, em parte por meio do trabalho desenvolvido pela Estratégia Saúde da Família (ESF) e com a participação intensiva dos agentes comunitários de saúde (ACS). Mesmo diante de alterações nas dinâmicas de seus territórios de atuação, das disparidades regionais, da heterogeneidade do processo de trabalho das equipes ^4^ e dos territórios marcados por vulnerabilidades sociais, como o aumento dos índices de violência em decorrência das desigualdades sociais acentuadas pela crise ^5^ ^,^ ^6^, os ACS atuaram na disseminação de informações sobre a pandemia e isolamento social, e na vigilância, com identificação e direcionamento de casos sugestivos e/ou confirmados da doença e busca ativa de novos casos ^7^ ^,^ ^8^.

Apesar de importante atuação, observou-se a sobreposição de tarefas e a fragilização da assistência à saúde prestada por esse profissional, especialmente quando atrelada à falta de infraestrutura das unidades de saúde, de capacitações e de insumos ^9^. O desgaste físico e mental resultante das dificuldades da organização do trabalho, da sobrecarga de funções e por vezes, da falta de apoio institucional para ações desenvolvidas, repercutiu em agravamento das condições de saúde dos ACS e do seu processo de trabalho ^9^. 

Apesar dos ACS mostrarem-se expostos às implicações de uma crise sanitária no seu cotidiano de trabalho e qualidade de vida, faz-se necessário avaliar a relação entre as implicações de uma crise, como a pandemia de COVID-19, no cotidiano laboral e pessoal desses profissionais à luz da categoria gênero, especialmente, em decorrência desta profissão ser composta principalmente por mulheres.

A força de trabalho na saúde é majoritariamente feminina e o papel social de gênero agrava as implicações de uma crise para as mulheres ^10^. A literatura revela, por exemplo, que mulheres da área da saúde reportam mais exaustão mental e manifestam mais sintomas da síndrome de Burnout que os homens ^11^.

Estudo conduzido na América do Norte ^12^, que avaliou o acometimento de médicas por fatores de estresse durante a pandemia de COVID-19, revelou que embora fatores pessoais combinados com aspectos organizacionais possuam implicações para a saúde das médicas, a emergência sanitária intensificou os efeitos de doenças físicas e mentais no gênero feminino. No Japão, uma avaliação dos efeitos da pandemia na vida de médicos demonstrou que mães médicas vivenciam dilemas entre a crescente demanda doméstica e as funções clínicas no hospital, em proporção significativamente maior do que a de pais médicos ^13^.

Tal disparidade perpassa a precarização do trabalho feminino no contexto da assistência à saúde. Neste cenário, o trabalho da profissional ACS é intrinsecamente associado ao trabalho doméstico feminino de “cuidado”, o que justifica e sustenta a continuidade de uma injusta divisão social e sexual do trabalho, que resulta, entre outros efeitos, em uma sobrecarga de trabalho - produtivo e reprodutivo - para a maioria das mulheres, particularmente às pertencentes à esta classe trabalhadora ^14^.

Assim, os impactos sanitários, econômicos e psicossociais decorrentes de uma crise sanitária podem evidenciar disparidades na prática laboral dos ACS, especialmente sob o recorte de gênero, que historicamente atravessa a vida das mulheres e que, em decorrência do contexto da pandemia, pode ter acentuado desigualdades.

Nesta perspectiva, o estudo apresenta como objetivo investigar a saúde mental, a qualidade de vida, o suporte social e as atividades de ACS durante e após uma crise sanitária, sob o recorte de gênero e de tempo decorrido. 

## Método

Estudo multicêntrico, longitudinal, de abordagem quantitativa, realizado na APS de diferentes municípios do Nordeste do Brasil, incluindo quatro capitais: Fortaleza (Ceará), João Pessoa (Paraíba), Recife (Pernambuco) e Teresina (Piauí), além de quatro cidades do interior do Ceará: Crato, Juazeiro do Norte, Barbalha e Sobral, nos anos de 2021 e 2023.

Os municípios lócus do estudo apresentam APS em processo de expansão e consolidação em suas bases territoriais. Na Paraíba, o Município de João Pessoa com 825.796 habitantes, possui 203 equipes de saúde da família (EqSF) e cobertura de APS de 87,04%. Em Pernambuco, Recife apresenta 1.661.017 habitantes, 333 EqSF e 71,67% de cobertura. No Piauí, Teresina possui 871.126 habitantes, 296 EqSF e 105,35% de cobertura. No Ceará, o Município de Fortaleza apresenta 2.669.342 habitantes, 466 EqSF e 61,34% de cobertura; Barbalha 59.732 habitantes, 26 EqSF e cobertura de 152,34%; Crato 132.123 habitantes, 46 EqSF e cobertura de 121,87%; Juazeiro do Norte 274.207 habitantes, 79 EqSF e 108,87% de cobertura e por fim, Sobral com seus 208.935 habitantes, possui 78 EqSF e 130,79% de cobertura de APS ^15^.

A pesquisa envolveu ACS selecionados aleatoriamente e convidados a participar do estudo, desde que estivessem ativos no trabalho por pelo menos um ano, e excluindo aqueles em férias ou licença médica nas duas ocasiões em que foram realizadas as coletas de dados.

O cálculo amostral aleatório simples para as coletas iniciais (2021 e 2023) adotou erro amostral de 5%, nível de 95% de confiança e distribuição homogênea (80/20) da população como parâmetro. O cálculo foi feito por município, utilizando-se como base a população de ACS registrada no *e-gestor* (Ministério da Saúde; https://egestoraps.saude.gov.br/) em 2020 (N = 7.909) e 2022 (N = 7.660), sendo a amostra calculada em 1.935 em 2021 e 1.915 ACS em 2023. 

Para coleta de dados em 2021 e 2023, procedeu-se treinamento da equipe de coletadores em todos os municípios, envolvendo instrumentos de coleta, protocolos de segurança frente à COVID-19 e técnicas de simulação (*role play*). Com anuência das Secretarias Municipais de Saúde e Comitê de Ética em Pesquisa (CEP), realizou-se levantamento e localização do quantitativo de unidades básicas de saúde (UBS) nos municípios e ACS a estas vinculados, sendo realizado sorteio aleatório para participação dos ACS, que foram contactados e receberam convites em suas unidades de vínculo. Termo de Consentimento Livre e Esclarecido (TCLE) foi assinado, sendo posteriormente a coleta realizada em local privativo na própria unidade. No ano de 2021, o período de coleta de dados ocorreu de abril a agosto e em 2023, de julho a novembro. 

Com o foco de analisar longitudinalmente as informações coletadas, os dados utilizados na presente pesquisa são referentes a ACS que participaram das duas coletas de dados. Para identificação e pareamento destes, utilizou-se o *propensity score matching* (PSM), com os ACS que responderam ao questionário em 2021 e em 2023. Para o PSM utilizou-se como parâmetros fixos: gênero, município de residência e unidade de saúde em que trabalha. Diferença de dois anos de idade entre a coleta de 2021 e 2023 também foi utilizada como parâmetro de pareamento. Para o pareamento utilizou-se o software estatístico R (http://www.r-project.org), o método *genetic*, *distance logit* e, para a diferença de idade, *caliper* de 0,2. 

Adotou-se um questionário estruturado elaborado como instrumento de coleta de dados com informações sociodemográficas, profissiográficas e referentes à violência (viu ou presenciou atos de violência [e.g., agressão verbal e ou física, assalto, esfaqueamento, tiro letal ou não-letal, estupro], se este agravo estava presente nos seus territórios de atuação, assim como se já havia sofrido violência no exercício de suas funções). 

Além deste, foram utilizados os seguintes instrumentos já validados na literatura: o *Questionário de Autorrelato* (*Self-Reporting Questionnaire*; SRQ-20), desenvolvido pela Organização Mundial da Saúde (OMS), é uma escala de rastreio e triagem para sofrimento mental, especialmente em grupos de trabalhadores, adotando um ponto de corte > 7 ^16^; o WHOQOL-BREF (*World Health Organization Quality of Life Abbreviated Instrument*) é um instrumento utilizado para a avaliação da qualidade de vida obtida por meio de quatro domínios (físico, psicológico, relações sociais e ambiente) ^17^ ^,^ ^18^; a *Escala de Autoeficácia Geral* ^19^ foi utilizada para medir a autoeficácia dos ACS, como a maior capacidade para controlar acontecimentos estressantes e resolver essas situações; a *Escala Multidimensional de Suporte Social Percebido* (*Multidimensional Scale of Perceived Social Support −* MSPSS) ^20^ ^,^ ^21^, objetivando-se conhecer o acesso de ACS aos recursos sociais disponíveis no enfrentamento de adversidades; e por fim, a *Escala de Ansiedade do Coronavírus* é utilizada para rastrear ansiedade relacionada à COVID-19 ^22^. Essa escala, desenvolvida na língua inglesa, possui cinco perguntas. Para sua utilização em português na presente pesquisa, realizou-se os seguintes passos: tradução, tradução em reverso, e validação de compreensão de português por um subgrupo de ACS.

Para descrever as características da amostra foram estimadas frequências absolutas e relativas das variáveis nominais, desvio padrão das variáveis contínuas e intervalos de 95% de confiança (IC95%). Analisou-se os dados longitudinalmente, observando se existia efeito do tempo e do gênero, assim como interação entre estes. Para as análises longitudinais, utilizou-se o banco de dados pareado (PSM) e análise estatística MANOVA (variáveis contínuas) e Q de Cochrane (variáveis categóricas). As comparações entre dois grupos foram realizadas utilizando teste t pareado (variáveis contínuas) e qui-quadrado (variáveis categóricas). Os testes estatísticos foram aplicados considerando o nível de 5% de significância. 

A coleta de dados da pesquisa realizada no ano de 2021 foi aprovada pelo CEP da Universidade Estadual do Ceará (UECE), com parecer 4.587.955, e a realizada no ano de 2023 aprovada pelo CEP do Instituto para Desenvolvimento da Educação (IPADE) do Centro Universitário Christus (Unichristus, parecer 5.917.599).

## Resultados

Participaram do estudo um total de 705 ACS (pareados pelo PSM) nos anos de 2021 (Tempo 1 - T1) e 2023 (Tempo 2 - T2). Em sua maioria, os ACS eram do sexo feminino (n = 598, 84,8%), apresentando no último ano de coleta (T2), idade média de 47,63 anos (DP ± 8,9 anos) e tempo médio de moradia no bairro de atuação de 30,58 anos (DP ± 15,17 anos), estado civil casado ou em união estável (n = 403, 57,33%) e de cor parda (n = 501, 71,57%). Quando se analisou os dados sociais e profissiográficos, observou-se o efeito do tempo na maioria das variáveis, como a elevação da escolaridade por meio da conclusão do Ensino Superior e aumento da renda salarial. O efeito do gênero foi identificado na idade dos participantes do estudo, sendo os homens mais novos que as mulheres.

Uma série de mudanças podem ser observadas em relação à violência, ao suporte social, à qualidade de vida e à saúde mental entre os anos de 2021 e 2023. De uma maneira geral, verifica-se aumento nas atividades desenvolvidas pelos ACS, diminuição da ansiedade vinculada ao coronavírus, redução do percentual de ACS com risco de transtornos mentais comuns (TMC), diminuição do suporte social e aumento na autoeficácia. 

Quanto às atividades desenvolvidas pelos ACS entre os tempos estudados, verificou-se aumento em sua variedade, com efeito significante do tempo na elevação da diversidade e quantidade de ações realizadas (p < 0,001). No que se refere à visita domiciliar, principal atividade desenvolvida pelo ACS, observa-se um aumento da realização de visitas por todas as motivações questionadas, especialmente para entrega de medicamentos, que passou de 37,93% (n = 267) em 2021 para 75,95% (n = 521) em 2023, com efeito significante do tempo (p < 0,001). Quanto ao efeito significante do gênero, observa-se que ACS do gênero masculino realizaram mais visitas domiciliares por demanda das famílias em 2021, bem como desenvolveram mais ações junto ao Programa Saúde na Escola (PSE) em ambos os anos, conforme Tabela 1. 

**Tabela 1:** Atividades desenvolvidas por agentes comunitários de saúde (ACS) durante e após a emergência sanitária da pandemia COVID-19; 2021-2023.

Características	2021 (T1) *				2023 (T2) *				Efeitos **		
	Feminino [n = 598]	Masculino [n = 107]	Valor de p	Total [n = 705]	Feminino [n = 598]	Masculino [n = 107]	Valor de p	Total [n = 705]	Gênero (valor de p	Tempo (valor de p)	G x T (valor de p)
Variedade de atividades desenvolvidas [média (DP)]	4,87 (1,39)	5,17 (1,12)	0,069	4,91 (1,35)	5,64 (0,74)	5,82 (0,60)	< 0,001	5,66 (0,73)	0,004	< 0,001	0,442
Variedade de tipos de visitas domiciliares realizadas [média (DP)]	4,86 (1,04)	4,97 (0,93)	0,400	4,88 (1,03)	5,61 (0,71)	5,56 (0,65)	0,200	5,61 (0,70)	0,670	< 0,001	0,190
Tipos de atividades realizadas [n (%)]											
Visitas domiciliares	592 (99,16)	107 (100,00)	> 0,900	699 (99,29)	587 (100,00)	107 (100,00)	-	694 (100,00)	-	0,073	-
Atividades de promoção da saúde com grupos específicos	490 (82,08)	92 (85,98)	0,300	582 (82,67)	568 (98,44)	105 (100,00)	0,400	673 (98,68)	-	< 0,001	-
Atividades do Programa Saúde na Escola	393 (65,83)	82 (76,64)	0,028	475 (67,47)	504 (90,32)	95 (96,94)	0,032	599 (91,31)	-	< 0,001	-
Atendimentos na comunidade	455 (76,21)	86 (80,37)	0,300	541 (76,85)	581 (98,14)	105 (100,00)	0,400	686 (98,42)	-	< 0,001	-
Atividade de acompanhamento da comunidade na unidade de saúde	530 (88,78)	98 (91,59)	0,400	628 (89,20)	588 (99,32)	105 (99,06)	0,600	693 (99,28)	-	< 0,001	-
Atividade burocrática na unidade de saúde	451 (75,54)	88 (82,24)	0,130	539 (76,56)	542 (95,25)	106 (99,07)	0,110	648 (95,86)	-	< 0,001	-
Tipos de visitas realizadas [n (%)]											
Por demanda das famílias	465 (77,89)	94 (87,85)	0,019	559 (79,40)	576 (100,00)	103 (100,00)	-	679 (100,00)	-	< 0,001	-
Para cadastro familiar, atualização do e-SUS	581 (97,32)	103 (96,26)	0,500	684 (97,16)	584 (100,00)	105 (100,00)	-	689 (100,00)	-	< 0,001	-
Busca ativa de casos	563 (94,30)	102 (95,33)	0,700	665 (94,46)	575 (100,00)	105 (100,00)	-	680 (100,00)	-	< 0,001	-
Programas específicos	560 (93,80)	101 (94,39)	0,800	661 (93,89)	587 (100,00)	103 (100,00)	-	690 (100,00)	-	< 0,001	-
Acompanhamento de visita domiciliar de profissionais de nível superior	509 (85,26)	94 (87,85)	0,500	603 (85,65)	587 (100,00)	106 (100,00)	-	693 (100,00)	-	< 0,001	-
Entrega de medicamentos	229 (38,36)	38 (35,51)	0,600	267 (37,93)	448 (76,98)	73 (70,19)	0,140	521 (75,95)	-	< 0,001	-

DP: desvio padrão; G x T: interação entre gênero e tempo; T1: tempo 1; T2: tempo 2.

Fonte: elaboração própria.

* Teste t pareado;

** MANOVA.

Assim, evidenciou-se que as atividades desenvolvidas pelos ACS sofreram influência do período pandêmico e pós. Neste contexto, a ansiedade associada ao coronavírus foi questionada, haja vista ser uma condição com potencial impacto nas atividades desenvolvidas por esse grupo. Neste quesito, observa-se a redução desta ansiedade com o tempo, passando de 0,72 (DP ± 0,90) em 2021 para 0,44 (DP ± 0,71) em 2023. Por outro lado, verificou-se elevação nos índices de autoeficácia dos ACS, que passou de 3,27 (DP ± 0,52) para 3,34 (DP ± 0,51) (Tabela 2).

**Tabela 2:** Saúde mental, qualidade de vida e apoio social de agentes comunitários de saúde (ACS) durante e após emergência sanitária da pandemia COVID-19, 2021-2023.

Variáveis	2021 (T1) *				2023 (T2) *				Efeitos **		
	Feminino [n = 598]	Masculino [n = 107]	Valor de p	Total [n = 705]	Feminino [n = 598]	Masculino [n = 107]	Valor de p	Total [n = 705]	Gênero (valor de p)	Tempo (valor de p)	G x T ** (valor de p)
Ansiedade do coronavírus [média (DP)]	0,77 (0,93)	0,48 (0,67)	0,004	0,72 (0,90)	0,46 (0,72)	0,34 (0,65)	0,120	0,44 (0,71)	0,002	< 0,001	0,102
SRQ-20 [média (DP)]	6,83 (4,85)	4,26 (4,61)	< 0,001	6,44 (4,90)	6,09 (4,80)	3,82 (4,20)	< 0,001	5,74 (4,78)	< 0,001	0,064	0,354
SRQ-20 grupos [n (%)]			< 0,001				< 0,001		-	0,002	-
Sem agravo em saúde mental	298 (49,83)	80 (74,77)	-	378 (53,62)	303 (57,39)	75 (78,13)	-	378 (60,58)	-	-	-
Sofrimento mental	300 (50,17)	27 (25,23)	-	327 (46,38)	225 (42,61)	21 (21,88)	-	246 (39,42)	-	-	-
MSPSS											
Família [média (DP)]	5,71 (1,43)	5,55 (1,37)	0,100	5,68 (1,43)	5,42 (1,44)	5,40 (1,42)	0,700	5,42 (1,43)	0,48	0,011	0,47
Amigos [média (DP)]	5,37 (1,41)	4,88 (1,37)	< 0,001	5,29 (1,42)	5,09 (1,31)	4,67 (1,38)	0,001	5,02 (1,33)	< 0,001	0,004	0,663
Outros significativos [média (DP)]	5,98 (1,27)	5,85 (1,43)	0,600	5,96 (1,29)	5,69 (1,26)	5,64 (1,28)	0,500	5,69 (1,27)	0,39	0,002	0,674
Escore total [média (DP)]	5,68 (1,18)	5,43 (1,14)	0,010	5,64 (1,17)	5,40 (1,14)	5,24 (1,18)	0,200	5,37 (1,15)	0,032	0,001	0,58
Autoeficácia geral [média (DP)]	3,27 (0,53)	3,27 (0,47)	0,800	3,27 (0,52)	3,34 (0,52)	3,33 (0,48)	0,400	3,34 (0,51)	0,876	0,031	0,736
WHOQOL											
Domínio físico [média (DP)]	3,39 (0,68)	3,57 (0,65)	0,003	3,42 (0,68)	3,61 (0,63)	3,74 (0,63)	0,084	3,63 (0,63)	0,007	< 0,001	0,502
Domínio psicológico [média (DP)]	3,77 (0,61)	3,92 (0,53)	0,017	3,79 (0,60)	3,63 (0,58)	3,70 (0,62)	0,300	3,64 (0,58)	0,026	< 0,001	0,248
Domínio relações sociais [média (DP)]	3,75 (0,77)	3,74 (0,66)	> 0,900	3,75 (0,76)	3,78 (0,67)	3,80 (0,70)	0,600	3,78 (0,67)	0,898	0,329	0,738
Domínio meio ambiente [média (DP)]	3,28 (0,59)	3,34 (0,54)	0,130	3,28 (0,58)	3,38 (0,53)	3,35 (0,58)	0,600	3,38 (0,54)	0,769	0,069	0,173
Escore total [média (DP)]	3,50 (0,53)	3,61 (0,48)	0,022	3,51 (0,52)	3,57 (0,49)	3,62 (0,52)	0,400	3,58 (0,49)	0,083	0,149	0,234

DP: desvio padrão; G x T: interação entre gênero e tempo; MSPSS: *Multidimensional Scale of Perceived Social Support*; SRQ-20: Questionário de Autorrelato (*Self-Reporting Questionnaire*); T1: tempo 1; T2: tempo 2; WHOQOL-BREF: *World Health Organization Quality of Life Abbreviated Instrument.*

Fonte: elaboração própria.

* Teste t pareado;

** MANOVA.

Quanto ao suporte social recebido, obtido por meio da aplicação do MSPSS, ao se questionar o papel da família como apoio, verifica-se que o tempo teve efeito significante, para ambos os gêneros, na redução do suporte familiar (p = 0,011). Frente ao suporte social recebido por amigos, gênero e tempo apresentaram significância, uma vez que se evidenciou redução deste suporte entre T1 e T2 (p = 0,004) de forma mais acentuada em ACS mulheres (p < 0,001), cenário este também encontrado frente ao escore geral do suporte social recebido, com efeito tempo (p = 0,001) e gênero (p = 0,032) significantes (Tabela 2).

A qualidade de vida foi outra variável investigada utilizando o WHOQOL em seus quatro domínios: físico, psicológico, social e ambiental. Observou-se elevação dos níveis relacionados ao domínio físico e diminuição no domínio psicológico da qualidade de vida nos ACS, sendo, em 2021, os índices destes domínios menores nas ACS do gênero feminino (Tabela 2). 

Outra condição questionada foi a violência nos territórios de atuação dos ACS, visto esta condição ter se agravado no período pandêmico. Embora não se observem diferenças no índice geral de violência (ter visto, saber sobre e/ou ter sofrido) nos anos estudados, os achados revelam elevação da exposição à violência pelos participantes durante sua prática laboral, que em 2021 registrou 40,20% (n = 283) de ACS vitimizados contra 51,07% (n = 357) em 2023. Interação entre gênero e tempo foi observada no índice de violência - aconteceu, onde a ocorrência de violência contra ACS do gênero feminino diminuiu com o tempo, enquanto a mesma aumentou para ACS do gênero masculino (Tabela 3).

**Tabela 3:** Violência e trabalho do agentes comunitários de saúde (ACS) durante e após emergência sanitária da pandemia COVID-19, 2021-2023.

Variáveis	2021 (T1) *			2023 (T2) *			Efeitos **		
	Feminino [n = 598]	Masculino [n = 107]	Valor de p	Total [n = 705]	Feminino [n = 598]	Masculino [n = 107]	Valor de p	Total [n = 705]	Gênero (valor de p)	Tempo (valor de p)	G x T ** (valor de p)
Índice de violência − viu/soube [média (DP)]	0,48 (0,33)	0,48 (0,31)	> 0,900	0,48 (0,33)	0,51 (0,36)	0,60 (0,36)	0,200	0,52 (0,36)	0,235	0,045	0,463
Índice de violência − aconteceu [média (DP)]	0,26 (0,28)	0,24 (0,28)	0,500	0,26 (0,28)	0,19 (0,23)	0,36 (0,40)	0,200	0,21 (0,26)	0,546	0,032	0,026
Índice de violência − geral [média (DP)]	0,37 (0,26)	0,36 (0,24)	> 0,900	0,37 (0,26)	0,33 (0,24)	0,45 (0,35)	0,200	0,35 (0,26)	0,112	0,260	0,212
A violência está presente na comunidade onde você atua? [n (%)]			0,900				> 0,900	-	ns	0,349	-
Não	149 (25,04)	26 (24,30)	-	175 (24,93)	137 (23,06)	24 (22,64)	-	161 (23,00)	-	-	-
Sim	446 (74,96)	81 (75,70)	-	527 (75,07)	457 (76,94)	82 (77,36)	-	539 (77,00)	-	-	-
Você já sofreu algum tipo de violência durante seu trabalho como ACS [n (%)]			0,400				0,700	-	-	< 0,001	-
Não	353 (59,13)	68 (63,55)	-	421 (59,80)	288 (48,57)	54 (50,94)	-	342 (48,93)	-	-	-
Sim	244 (40,87)	39 (36,45)	-	283 (40,20)	305 (51,43)	52 (49,06)	-	357 (51,07)	-	-	-

DP: desvio padrão; G x T: interação entre gênero e tempo; ns: não se aplica; T1: tempo 1; T2: tempo 2.

Fonte: elaboração própria.

* Teste t pareado;

** MANOVA.

## Discussão

Este manuscrito investigou o efeito do gênero e do tempo em uma série de questões relacionadas aos ACS em dois períodos distintos: a pandemia de COVID-19 (1º semestre de 2021) e após a emergência pandêmica (2º semestre de 2023). Entre as questões investigadas tem-se o perfil sociodemográfico e profissional, violência (índice de violência), suporte social, qualidade de vida, saúde mental e processo de trabalho. Evidenciaram-se várias diferenças entre estes dois períodos, algumas com efeito do tempo, outras com efeito do gênero e ainda, com interação entre gênero e tempo.

É nítido que a pandemia de COVID-19 demandou reestruturação dos sistemas de saúde e reorganização dos processos de trabalho e fluxos assistenciais em todos os níveis de atenção, como na APS. Neste sentido, a reorganização do processo de trabalho do ACS se fez necessária, objetivando otimizar as ações desenvolvidas, embora tenha se evidenciado aumento da carga de trabalho deste profissional durante e após a pandemia ^23^.

Neste cenário, se observa aumento da realização de visitas domiciliares e outras atividades no período pós emergência sanitária, sendo a entrega de medicamento o único tipo que não totaliza 100%. O acesso e acompanhamento às pessoas/famílias em situação de vulnerabilidade se caracteriza como a principal atribuição do ACS por meio da visita domiciliar, contribuindo para melhor acesso, continuidade do cuidado e fortalecimento de vínculos entre profissionais e usuários ^24^.

Ainda, ações vinculadas ao PSE, atendimentos na comunidade e ações burocráticas/administrativas foram as que mais se elevaram ao longo do tempo nesta pesquisa. Durante o período pandêmico, evidenciou-se elevação na diversidade de funções e atividades desenvolvidas pelos ACS ^25^. O redirecionamento de ações deste profissional surge como resposta do Governo Federal para a reorganização do trabalho dos ACS, permeado por racionalidades utilitaristas, com foco no trabalho administrativo, em conformidade com normativas identificadas nos últimos anos, exacerbadas pela pandemia ^26^.

É notável que o aumento da carga de trabalho associado ao medo da contaminação e exaustão emocional contribuíram para a intensificação de problemas emocionais entre ACS ^27^. Pesquisa desenvolvida por De Boni et al. ^27^ durante a pandemia, revelou que ansiedade e depressão afetaram 47,3% dos trabalhadores de serviços essenciais, entre estes os profissionais da saúde. Tal resultado corrobora com achados obtidos neste estudo, ao revelar que a ansiedade oriunda do cenário pandêmico esteve presente entre os ACS e se somou ao cotidiano de trabalho deste profissional, repercutindo nas suas funções.

Entre todos os profissionais que atuam na APS, há maior incidência de ansiedade e depressão entre ACS ^28^, o que aponta um cenário preocupante em relação à saúde emocional desses trabalhadores. A presença de sofrimento mental e transtornos psíquicos nos ACS é comum quando estes têm que lidar com situações que causam sofrimento no ambiente de trabalho ^29^. A falta de condições adequadas para o exercício da função, déficits de estrutura física e mau relacionamento com a população/equipe/gestores influenciam as condições psicoemocionais destes profissionais ^28^, sendo razoável supor que isso se agravaria em uma situação de crise, como a pandemia.

Os dados desta pesquisa corroboram com achados de inquérito realizado com profissionais de saúde no Município do Rio de Janeiro. Dos 2.996 investigados, cerca da metade apresentava grau leve, moderado ou severo de TMC, como depressão, ansiedade ou estresse ^30^. Em Minas Gerais, maiores índices de depressão e ansiedade foram identificados entre ACS durante a pandemia, prioritariamente naqueles com idade até 40 anos, mais de 500 usuários cadastrados sob sua responsabilidade e com inadequada oferta de equipamentos de proteção individual (EPI) ^31^. 

De um modo geral, a pandemia impôs aos profissionais de saúde alterações no ambiente de trabalho e excesso de demandas que, em conjunto, elevaram as atribuições profissionais e se somaram às preocupações com o risco de infecção (própria e de familiares), adoecimento e morte. As particularidades do trabalho posicionaram esses profissionais em situações de risco (saúde física e mental), com alta insegurança laboral ^26^. Este cenário ocasiona sofrimento psíquico, manifestados por meio de transtornos de ansiedade, distúrbios do sono, depressão, medo de adoecimento e contaminação, fadiga, irritabilidade, esquecimento, dificuldades de concentração e queixas somáticas ^32^, evidenciados no presente estudo pelo maior valor do SRQ-20 durante a pandemia (2021).

Destaca-se que problemas relacionados à saúde mental foram mais evidentes nas ACS mulheres em 2021, corroborando com estudos da população geral no Brasil durante a pandemia, onde mulheres apresentaram maior incidência de sintomas de ansiedade e depressão ^33^. Estudos realizados nas Filipinas, China e Itália apontaram a relevância do gênero como preditor de sofrimento mental durante a COVID-19 ^34^ ^,^ ^35^ ^,^ ^36^, confirmando as mulheres como o grupo com maior risco para efeitos psicológicos adversos durante uma crise de saúde pública ^34^ ^,^ ^35^. 

As disparidades de gênero na sociedade reforçam a predominância de mulheres na área da saúde. Esta inclinação pode estar ligada a aspectos culturais relacionados à profissão, que se volta para o cuidar, compreendida como uma ocupação destinada às mulheres, devido à ligação com o zelo social que essas atividades envolvem ^37^, o que gera sobrecarga de tarefas e sofrimento mental. Assim, é possível que transtornos mentais possam ser considerados produtos de uma violência de gênero, resultante da divisão sexual do trabalho que relega às mulheres o exercício do cuidado. Durante a pandemia, esta violência produziu sobre as mulheres duplas jornadas, excesso de responsabilização e violências no cotidiano de trabalho, impactando no adoecimento mental nestas profissionais ^38^.

Apesar do elevado risco para TMC registrados pelos participantes do estudo durante a pandemia, verificou-se sua redução com o tempo, especialmente no grupo feminino, o mais afetado, corroborando com inquérito que revelou diminuição nos níveis de depressão e ansiedade da população entre a primeira e a quarta onda de COVID-19 no Brasil ^39^.

A presença de uma rede de suporte é importante para o enfrentamento deste cenário, sendo tal condição relatada pelos participantes. A família e o círculo de amizades despontaram como fontes de suporte social recebido durante a pandemia, com destaque para ACS mulheres, mais impactadas emocionalmente. É observado maior apego de mulheres às suas famílias de origem como resultado da socialização do gênero, já que muitas vezes estas são ensinadas a cuidar dos familiares ^40^. Adicionalmente, as mulheres, em geral, sentem uma necessidade maior de se relacionarem com outras pessoas, o que pode justificar, neste estudo, maior contato e suporte social recebido de familiares e amigos. 

É esperado da família o cuidado em caso de incapacidade ou adoecimento, sendo este o vínculo primeiro a ser acionado ^41^. Assim, o suporte social percebido pela família refere-se à avaliação subjetiva de que existe apoio disponível, caso dele se necessite ^42^, como por exemplo, em tempos de crise.

Desta forma, é possível que a urgência do cenário pandêmico tenha levado a uma reelaboração das relações familiares e, mesmo diante da impossibilidade de contato físico, os contatos virtuais foram fortalecidos ^43^. A proximidade com entes queridos é reconhecida com fonte positiva de apoio diante de momentos difíceis, minimizando estresse e ansiedade ^44^.

Pesquisa conduzida com profissionais de saúde da linha de frente ao coronavírus na China, revelou que o apoio familiar foi relevante para promover o bem-estar do profissional, diminuindo sintomas de ansiedade e depressão ^45^. No Reino Unido, indivíduos com diagnósticos de transtornos mentais já existentes apresentavam maior probabilidade de experimentarem sentimentos intensos de solidão. No entanto, aspectos sociais, como conviver com outras pessoas, contar com amigos próximos e perceber apoio social contribuíram para proteção contra esse fenômeno ^46^.

Embora fosse muito importante durante os momentos de crise, com o término da crise pandêmica, verificou-se diminuição do suporte social, e aumento da autoeficácia dos ACS. 

Sabe-se que diante de um cenário pandêmico, é possível que sintomas de TMC e outros transtornos psicológicos imponham desafios cotidianos que podem afetar negativamente a confiança para o cumprimento de tarefas e redução da autoeficácia. Relação entre baixa autoeficácia e ansiedade em enfermeiros foi observada no contexto da pandemia de COVID-19 ^47^. É esperado que, com a redução do sofrimento mental, haja maior motivação, segurança, crédito e competência no desempenho de atividades. Um forte senso de competência facilita os processos cognitivos, o desempenho e o enfrentamento de situações adversas, que torna o indivíduo menos vulnerável aos TMC ^47^.

A crença na autoeficácia e outras variáveis contribuem para a autorregulação do comportamento ^47^ e este, associado a hábitos de vida, são considerados importantes para a avaliação da qualidade de vida. Neste estudo, observou-se elevação dos níveis relacionados aos domínios físico e psicológico da qualidade de vida nos ACS, sendo, em 2021, os índices destes domínios menores nas ACS do gênero feminino. 

Segundo a Organização das Nações Unidas para a Igualdade de Gênero e o Empoderamento das Mulheres ^48^, diante da saturação dos sistemas de saúde e fechamento das escolas, as mulheres passaram a ser as mais afetadas devido à sobrecarga de trabalho, dedicando-se ao cuidado de familiares e tarefas domésticas. No caso das mulheres do setor saúde, além de assumirem responsabilidade profissional diante da pandemia, continuaram sendo as principais cuidadoras em domicílio, sobrecarregando-se duplamente ^49^. Estes dados corroboram com os achados deste estudo, ao demonstrar menores índices dos domínios físico e psicológico entre as ACS no período pandêmico.

O domínio físico da qualidade de vida inclui questões relacionadas à energia, sono, mobilidade, atividades da vida diária e capacidade para o trabalho e o domínio psicológico contempla questões voltadas à pensamentos/sentimentos, capacidade de concentração, autoestima e imagem corporal ^50^. Infere-se que a redução nos indicadores de morbimortalidade da COVID-19 e a não mais necessidade de isolamento social tenham impactado melhorias nesses domínios ao longo do tempo.

Contudo, dados deste estudo revelam que houve elevação dos índices de violência nos territórios de atuação dos ACS, com aumento de vitimização no exercício da função, mesmo com redução dos indicadores da pandemia de COVID-19 ao longo do tempo. A elevação da ocorrência de episódios violentos pode estar relacionada ao agravamento das desigualdades sociais decorrente das medidas de isolamento social, que culminaram em um cenário com baixo crescimento, aumento da pobreza, recessão e crescentes tensões sociais ^51^. Estas condições se constituíram cenário propício para o fortalecimento da violência urbana, especialmente em locais já vulnerabilizados, como aqueles aos quais estão inseridos os serviços da ESF, por priorizarem regiões com maior precariedade social ^5^.

Embora para ambos os gêneros, a violência tenha se elevado, ter sofrido violência no território de trabalho, em algum momento durante o período do estudo, apresentou-se em ascensão na população ACS masculina, com redução na feminina. Tal situação pode ter relação com maior reconhecimento e atuação em casos de violência por parte dos ACS homens. Estudo realizado com 1.239 ACS inseridos na APS do Município de Fortaleza, revelou que ACS do gênero masculino e aqueles com maior idade se sentem mais preparados para lidar com a violência ^5^.

Apesar de apresentar achados importantes, essa pesquisa possui como limitação estar vinculado a municípios da Região Nordeste, o que não permite a generalização dos achados, considerando os diversos contextos brasileiros e suas singularidades.

## Considerações finais

As repercussões da pandemia de COVID-19 se deram nos contextos pessoal e profissional do ACS, estando a ACS mulher mais vulnerável aos impactos negativos decorrentes da pandemia, refletindo a divisão social e sexual do trabalho presente no setor saúde.

Embora se evidencie melhorias na saúde mental, autoeficácia e qualidade de vida dos ACS do estudo no período pós pandemia, a sobrecarga de trabalho depositada no ACS durante a pandemia permanece. Portanto, faz-se necessário acompanhar estes profissionais, oferecendo apoio psicológico e gerenciando políticas públicas que possibilitem direcionar o processo de trabalho, considerando as repercussões individuais da pandemia e as disparidades de gênero nos espaços laborais.

Para pesquisas futuras, sugere-se a realização de estudos empíricos a partir de entrevistas com ACS, objetivando-se o aprofundamento das questões aqui levantadas, a fim de ampliar o entendimento acerca da dinâmica multidimensional que envolve violência nos territórios, sofrimento mental, suporte social e qualidade de vida de ACS sob a ótica das relações de gênero.
